# The Involvement of Angiotensin Type 1 and Type 2 Receptors in Estrogen-Induced Cell Proliferation and Vascular Endothelial Growth Factor Expression in the Rat Anterior Pituitary

**DOI:** 10.1100/2012/358102

**Published:** 2012-04-26

**Authors:** Hanna Lawnicka, Dorota Ptasinska-Wnuk, Slawomir Mucha, Jolanta Kunert-Radek, Marek Pawlikowski, Henryk Stepien

**Affiliations:** ^1^Department of Immunoendocrinology, Medical University of Lodz, Dr. Sterling 3 Street, 91-425 Lodz, Poland; ^2^Department of Endocrinology, The County Hospital of Kutno, 52 Kosciuszki Street, 99-300 Kutno, Poland; ^3^Clinic of Endocrinology, Medical University of Lodz, Dr. Sterling 3 Street, 91-425 Lodz, Poland

## Abstract

The aim of our study was to examine the involvement of renin-angiotensin system (RAS) in estrogen-induced lactotropes proliferation and vascular endothelial growth factor (VEGF) expression in rat pituitary. The study was performed on Fisher 344 rats underwent 8-day treatment with diethylstilboestrol (DES). The proliferation index (PCNA) and VEGF expression in pituitary sections were estimated using immunohistochemical methods. Treatment with DES increased the number of PCNA-positive cells, VEGF-positive cells, and VEGF-positive blood vessels in pituitary. Stimulatory effect of estrogen on cell proliferation and VEGF expression in blood vessels was attenuated by losartan, PD123319, and captopril. VEGF immunoreactivity in pituitary cells of DES-treated rats was decreased by AT1 antagonist and not changed by AT2 blocker and ACE inhibitor. Our findings suggest the involvement of RAS in DES-induced cell proliferation and VEGF expression in pituitary. Both the AT1 and AT2 receptors appear to mediate the estrogen-dependent mitogenic and proangiogenic effects in rat pituitary.

## 1. Introduction

Estrogens are well known to stimulate, both directly and indirectly, the cellular proliferation in anterior pituitary, and their growth-promoting effects are largely confined to the lactotropes [1–3]. Pituitary lactotropes hyperplasia has been observed in physiological states of estrogen excess, during estrus in several species, and in human pregnancy [4]. Heaney et al. have revealed the cyclic expression of the pituitary tumor transforming gene (*pttg*), basic fibroblast growth factor (bFGF), and vascular endothelial growth factor (VEGF) during the rat estrus cycle, with maximal expression occurring with peak serum estradiol (E2), increased pituitary proliferation and angiogenesis [5]. The functional role of estrogen receptor (ER*α*) in cell proliferation, prolactin (PRL) secretion, and expression of some growth factors, including the above-mentioned ones, in pituitary prolactinomas was documented as well [6]. E2 has been found to induce the transforming growth factor-beta 3 (TGF-*β*3) secretion from lactotropes. TGF-*β*3 stimulated, in turn, the folliculostellate (FS) cells to release both VEGF and bFGF, the potent mitogen for prolactin- (PRL-) secreting cells [7]. E2 augmented also a level of the key potent proangiogenic cytokine, VEGF, in *prolactinoma* tumor cell preparations [8]. *In vivo*, chronic treatment with estrogens resulted in the increased VEGF production and VEGF receptor expression in anterior pituitary of rats [9]. Moreover, tumor formation in estrogen-induced *prolactinoma* was shown to be associated with the development of the direct arterial blood supply leading the anterior pituitary cells to escape from hypothalamic control [10]. All these findings imply the pivotal role of estrogens in the molecular events responsible for lactotrope transformation and following prolactin-secreting adenoma development.

Renin-angiotensin system (RAS) is an essential regulator of the cardiovascular homeostasis and other fundamental biological functions, including hormonal secretion and cellular growth [11, 12]. Besides the circulating RAS, many organs and tissues are capable of producing angiotensin peptides [13–16]. All the components of RAS have been identified also within pituitary gland, and the main bioactive peptide of RAS, angiotensin II (ang II), has been proved to be synthesized intrapituitary [17]. There are many reports on the interaction between local tissue RAS and estrogens. In the anterior lobe of rat pituitary, the expression of angiotensin receptors (AT) fluctuated during estrous cycle, with the highest binding in diestrus and lowest binding in estrus [18]. Moreover, the number of AT1 receptor subtype and the activity of angiotensin-converting enzyme (ACE) in anterior pituitary of ovariectomized (OVX) rats fell after chronic treatment with E2 [19, 20]. Nevertheless, long-term exposition of lactotropic cells to the synthetic estrogen, diethylstilboestrol (DES), enhanced the AT1-dependent PRL secretion and strengthened the intracellular calcium mobilization and inositol phosphate generation in response to ang II, suggesting the stimulatory effects of estrogens on the AT1-linked postreceptor mechanisms [21, 22]. Estrogens were also shown to increase the plasma angiotensinogen level and to stimulate the expression of angiotensinogen mRNA in various tissues, including the pituitary gland [23]. Additionally, the release of ang II from hypothalamus has been described to rise in response to estrogen [24]. Next, a chronic exposition to DES led to the upregulation of the AT2 receptor gene transcription and increase in the functional AT2 receptor expression in anterior pituitary of rats [25].

Increasing lines of evidence indicate the important role of RAS in regulation of the cellular growth and angiogenesis within pituitary gland. Ang II and its derivative, ang IV, were found to exert the stimulatory effects on the anterior pituitary cell proliferation both *in vitro* and *in vivo* [26–28]. These peptides stimulated also the activity of tyrosine kinase (TK) in cells isolated from the estrogen-induced rat pituitary tumors [29, 30]. In lactosomatotroph GH3 cell culture, ang II, ang III, and ang IV were shown to affect the VEGF secretion [31]. Moreover, local or systemic RAS was suggested to mediate the estrogen-induced vascular changes in the anterior pituitary gland in rodents [52].

The goal of the present study was to investigate the potential involvement of RAS in estrogen-induced cellular proliferation and angiogenesis in anterior pituitary of rats. We performed the experiments using a high estrogen-responsive rat strain, Fisher 344 (F344). F344 is especially susceptible to the estrogens' growth promoting and tumor-inducing effects on the pituitary, as chronic treatment of rat with E2 leads to the lactotrope proliferation and following lactotropic tumor formation within a few months [32].

## 2. Material and Methods

### 2.1. Animals

Four-week-old male Fisher 344 rats, weighing ~60 g each were used in the experiment. The animals were kept in the controlled conditions of light/darkness (12 h/12 h) and temperature (23 ± 2°C) with standard laboratory food and water available ad libitum.

All procedures were approved by The Local Animal Use and Care Committee (no. Ł/BD/126).

### 2.2. Induction of Pituitary Cells Hyperplasia

The animals underwent chronic oestrogen treatment using the silastic capsules prepared from 5 mm long Silastic tubes of 1.57 mm inner diameter and 2.4 mm outer diameter (Silastic Laboratory Tubbing, Dow Corning Corporation, USA). These tubes were filled with a saturated solution of diethylstilboestrol (DES; Stilboestrolum, Polfa, Poland) in 96% ethanol. After evaporation of alcohol, the capsules, containing 8–10 mg of DES each, were sealed with silastic medical adhesive (Silastic Medical Adhesive Silicone Type A, Dow Corning Corporation, USA), protected from light and stored at 4°C. Such implants were estimated to release 18–45 *μ*g/day of DES [1] and to induce the massive hyperplasia of prolactin cells in the strain of rats used in the study.

To induce pituitary lactotropes hyperplasia, the animals underwent the intraperitoneal (i.p.) ketamine (Ketanest, Parke-Davis) anaesthesia (60 mg/kg of body weight) following the subcutaneous (s.c.) implantation of silastic capsules in lumbar region of each rat.

### 2.3. Experimental Protocol and Preparation of Tissues

Three days after capsules implantation, animals were divided into 5 groups (7 rats in each group) and started receiving examined substances as follows:

- Group I (control group without capsules; DES−): 0.5 mL 0.9% NaCl, i.p.;
- Group II (control group with capsules; DES+): 0.5 mL 0.9% NaCl, i.p.;
- Group III: Losartan (Merck), 10 mg/kg of body weight, i.p.;
- Group IV: PD123319 (PD123319 di(trifluoroacetate), Sigma), 1 mg/kg of body weight, i.p.;
- Group V: Captoril (Sigma), 50 mg/kg of body weight, s.c.

Injections were made twice a day (every 12 h) for the following 6 days. Twelve hours after the last injections, all animals were decapitated, pituitary glands were carefully isolated, fixed in 4% neutral buffered formalin and embedded in paraffin. The 5 *μ*m thick sections of the pituitary glands were mounted on normal glass slides for immunohistochemistry.

### 2.4. Immunohistochemistry

#### 2.4.1. Cell Proliferation Study

The determination of the proliferating cell nuclear antigen (PCNA) was applied as an index of cell proliferation. Immunohistochemistry was performed using a PC 10 monoclonal antibody (Monoclonal Mouse Anti-Rat Proliferating Cell Nuclear Antigen, Clone PC 10, DakoCytomation). After wax removal, sections were heated in citrate buffer (pH 6.0) in a microwave oven (700 W, 10 min) then chilled at room temperature and washed with distilled water and in Tris/HCl buffer (pH 7.4). Sections were then exposed to anti-PCNA PC 10 antibody for 10 minutes and washed again. The visualization of the reaction was achieved using EnVision System-AP (DakoCytomation). The protocol included incubation with Labelled Polymer for 10 min following washing procedure, incubation with Fast Red solution for 25 min, and subsequent washing with distilled water. The sections were then stained with hematoxylin for 45 min.

#### 2.4.2. VEGF Expression Study

VEGF expression in the anterior pituitary gland cells and vessels was determined using mouse monoclonal antibody IgG_2*α*_ (VEGF (C-1), Santa Cruz Biotechnology). After wax removal, sections were heated in citrate buffer (pH 6.0) in a microwave oven (700 W, 5 min) then chilled at room temperature, washed with PBS and incubated with 3% hydrogen peroxide (H_2_O_2_ solution) to block the endogenous peroxidase. The sections were then incubated with anti-VEGF (C-1) immunoglobulin at a dilution of 1 : 100 for 5 min. To intensify the reaction, StreptABComplez/HRP Duet, Mouse/Rabbit (DakoCytomation) kit was used. The protocol included incubation with anti-mouse-IgG_2*α*_ antibodies labeled with peroxidase (POD) for 30 min, washing with PBS, incubation with tetrachloride 3,3′-diaminobenzidine (DAB) solution for 4 min (POD substrate), and washing with running water. The sections were then stained with hematoxylin for 60 s.

#### 2.4.3. Image Analysis

Digital images were acquired from the light microscope (Olympus BX40, Japan) at ×200 magnification via CCD colour camera (CC20P; Videotronic GmbH, Germany) and analyzed using a computer system (MultiScanBase v.8.08; Computer Scanning System, Warsaw, Poland).

*The PCNA labeling index (LI)* was assessed as a number of PCNA-immunopositive nuclei per 1000 randomly scored anterior pituitary cells in microscopic preparations at 600x magnification. Minimum 4,000 cells were counted in each slice.

*VEGF-immunopositive anterior pituitary cells* were assessed as a number of VEGF-immunopositive cells per 1000 randomly scored cells in microscopic preparations at 600x magnification. Moreover, the VEGF-immunopositive cells were classified into one of three categories: the cells with weak, medium, or strong VEGF immunoreactivity. Minimum 4,000 cells were counted in each slice.

*VEGF-immunopositive reaction in anterior pituitary vessels *was expressed as the mean number of VEGF-immunopositive vessel sections per microscopic image, counted from 10 microscopic images, at 200x magnification.

### 2.5. Statistical Evaluation

The results are expressed as means ± SEM. Comparisons of individual groups were evaluated by analysis of variance (ANOVA) following LSD (least difference test) Fisher test. Differences were significant if *P* < 0.05.

## 3. Results

**Figure 1:**
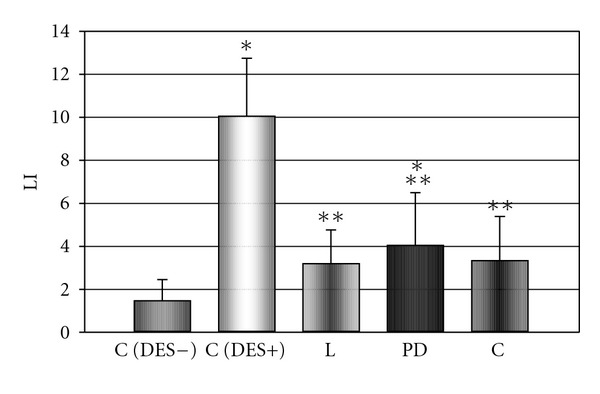
The influence of AT1 receptor antagonist losartan (L), AT2 receptor antagonist PD123319 (PD), and ACE inhibitor captopril (C) on diethylstilboestrol- (DES-) induced increase in number of PCNA immunopositive cells in anterior pituitary of rats. X ± SEM; *P < 0.05 versus C (DES−, control DES-untreated group), **P < 0.05 versus C (DES+, control DES-treated group), LI: labeling index (the number of PCNA-immunopositive cells per 1000 randomly scattered cells of anterior pituitary).

**Figure 2:**
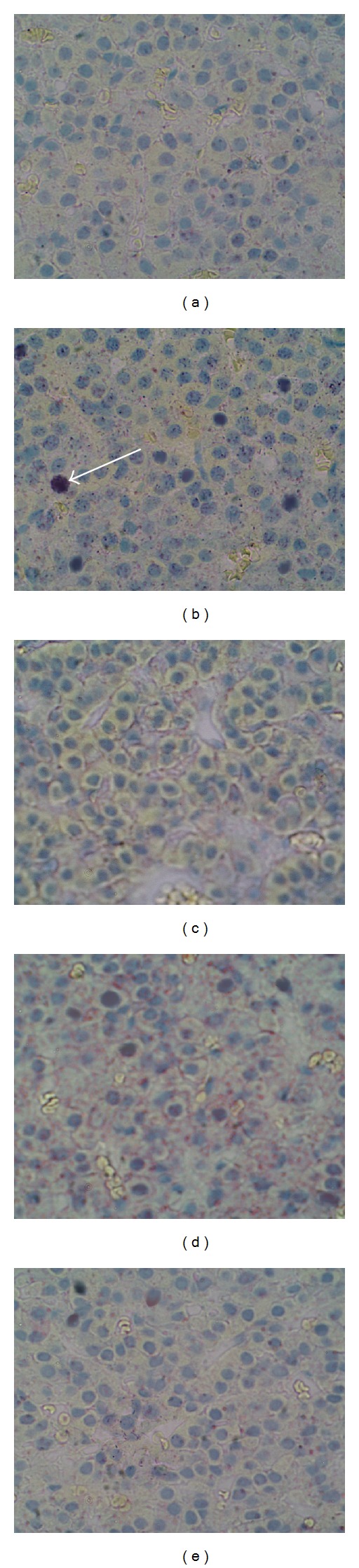
Immunohistochemical staining for the proliferation cell nuclear antigen (PCNA) in anterior pituitary of rats; (a) control diethylstilboestrol- (DES-) untreated group, (b) control DES-treated group, (c) DES and AT1 blocker (losartan) cotreated group, (d) DES and AT2 blocker (PD12319) cotreated group, and (e) DES and angiotensin converting enzyme inhibitor (captopril) cotreated group. White arrow indicates PCNA-positive cell.

**Figure 3:**
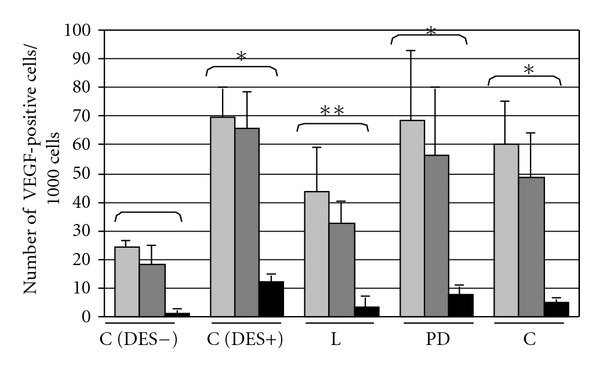
The influence of AT1 receptor antagonist losartan (L), AT2 receptor antagonist PD123319 (PD) and ACE inhibitor captopril (C) on diethylstilboestrol- (DES-) induced increase in number of vascular endothelial growth factor (VEGF) immunopositive cells in anterior pituitary of rats. X ± SEM; *P < 0.05 versus C (DES−, control DES-untreated group), **P < 0.05 versus C (DES+, control DES-treated group). pale gray: weak VEGF immunoreactivity; dark gray: medium VEGF immunoreactivity; black: strong VEGF immunoreactivity.

**Figure 4:**
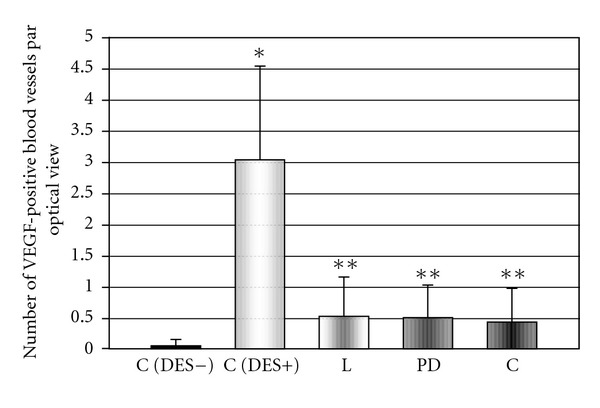
The influence of AT1 receptor antagonist losartan (L), AT2 receptor antagonist PD123319 (PD) and ACE inhibitor captopril (C) on diethylstilboestrol- (DES-) induced increase in number of VEGF immunopositive blood vessels in anterior pituitary of rats. X ± SEM; *P < 0.05 versus C (DES−, control DES-untreated group), **P < 0.05 versus C (DES+, control DES-treated group).

**Figure 5:**
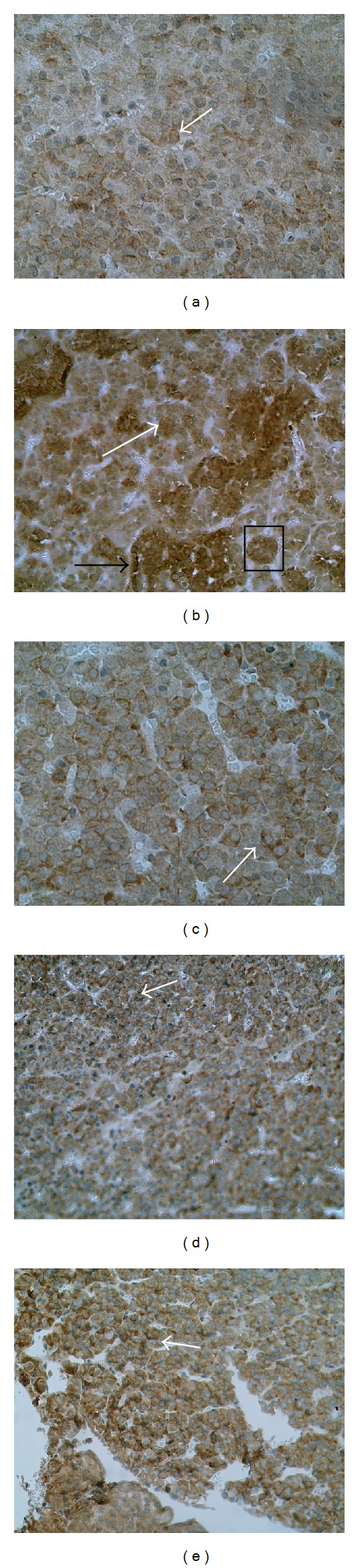
Immunohistochemical staining for vascular endothelial growth factor (VEGF) in anterior pituitary of rats; (a) control diethylstilboestrol- (DES-) untreated group, (b) control DES-treated group, (c) DES and AT1 blocker (losartan) cotreated group, (d) DES and AT2 blocker (PD12319) co-treated group, and (e) DES and angiotensin converting enzyme inhibitor (captopril) cotreated group. The white arrows indicate the single-VEGF-positive pituitary cell, the black arrow indicates the VEGF immunopositive blood vessel, within the black rectangular: the example isle of VEGF immunopositive cells in rat anterior pituitary.

The results of the quantitative analysis and the statistical evaluation of these results are presented in Figures 1–5.

The 8-day treatment with DES resulted in a significant elevation in the proliferation index, expressed as a number of the PCNA-immunopositive cells per 1000 randomly scattered cells, in anterior pituitary of rats (Figures 1 and 2(b)). At the same time, the number of PCNA-positive cells was significantly decreased in anterior pituitary of animals receiving the AT1 antagonist losartan, AT2 receptor antagonist PD123319 or ACE inhibitor captopril, comparing to the DES-treated control group (Figures 1, 2(c), 2(d), and 2(e)). These data indicate the inhibitory effects of RAS antagonists on the estrogen-dependent pituitary cell proliferation.

Concomitantly, exposition of rats to DES led to the increased VEGF immunoreactivity in anterior pituitary cells and blood vessels (Figures 3, 4, and 5(b)). Administration of losartan, PD123319, or captopril resulted in a significant decrease in the number of VEGF-positive blood vessels. (Figures 3, 5(c), 5(d) and 5(e)). The number of anterior pituitary cells staining for VEGF diminished in animals receiving only the AT1 antagonist and changed after co-treatment with neither AT2 blocker nor ACE inhibitor (Figures 4, 5(c), 5(d) and 5(e)).

## 4. Discussion

Pituitary gland is the source of various growth factors and cytokines that regulate cellular growth in an autocrine and paracrine manner [33]. Immunohistochemical studies have demonstrated that anterior pituitary cells are also able to express the components of RAS [34, 35]. At the other hand, angiotensin peptides have been shown to affect the growth of anterior pituitary cells. Ang II and ang IV increased cellular proliferation in the primary culture rat lactotropes [26]. These angiotensin peptides enhanced also BrdU incorporation in anterior pituitary of the ovariectomized female rats* in vivo* [27]. Furthermore, RAS has been suggested to mediate the estrogen-induced lactotropic cell proliferation and pituitary hyperplasia in rats [28].

 In agreement with the previous reports, chronic exposure to DES significantly increased the number of proliferating cells in anterior pituitary of rats. This mitogenic effect of estrogen was abrogated by the ACE inhibitor, captopril. Moreover, PCNA immunoreactivity was also significantly depressed in pituitaries of animals receiving the AT1 receptor antagonist losartan or AT2 receptor antagonist PD123319, comparing to the DES-treated control group. These findings corroborate with the earlier results concerning the effects of sex steroids on PRL release. Suarez et al. revealed that both the AT1 and AT2 receptor blockers inhibited the ang-II-induced secretion of PRL in rats underwent to the combined treatment with DES and progesterone [25]. Surprisingly, in previous *in vivo* study, neither losartan nor PD123319 decreased the cellular proliferation in DES-induced PRL-secreting tumor in rat, although it was inhibited by angiotensin convertase inhibitor, enalaprilate [28]. This discrepancy may be possibly explained by the resistance of *prolactinoma* cells to the growth-regulating factors. This is highly probable, that following 12-week exposure to DES, rat pituitary lactotropes have become autonomous, and thus insensitive to the endogenous angiotensin peptides affecting the growth of unstimulated cells. In the present study, we underwent animals to the only 8-day treatment with DES, thus attempting to prevent the development of such a pituitary cell autonomy. We believe that our experimental model has led us to examine the estrogen-stimulated, but still susceptible to the growth factors anterior pituitary cells, at the initial stages of the estrogen dependent tumorogenesis.

 AT1 receptor is well established to be essential for the mitogenic effects of ang II in various tissues and organs [36–41]. Moreover, blockade of AT1 signaling reduced tumor growth, angiogenesis, and metastasis in the model of murine sarcoma and fibrosarcoma cells [42]. In contrast, AT2 receptor has been often demonstrated to counteract the AT1-dependent proliferogenic actions [43, 44]. However, ang-II-induced apoptosis of the rat cardiomyocytes was connected with activation of both the AT2 and AT1 receptors [45]. Moreover, AT2 caused constitutive, ligand-independent growth and did not antagonize AT1-mediated cardiomyocytes hypertrophy [46]. Each of these receptors mediated the stimulatory effects of ang II on cellular proliferation and apoptosis in renal glomerular epithelial tissue as well [47]. Such a cooperation of the AT1 and AT2 receptors has been also demonstrated in our present study, as both receptors appeared to mediate the estrogen-induced proliferation of rat anterior pituitary cells.

Pituitary adenoma cells are able to produce the potent angiogenic factor, VEGF, which is possibly involved in the intratumoral blood vessel formation. VEGF was shown to be present in various pituitary tumor cell lines, in primary cultures of pituitary adenomas and in fragments from the human pituitary adenomas obtained at surgery [8, 48]. The production of this cytokine in lactosomatotroph GH3 cells and in *prolactinoma* tumor cell preparations was stimulated by estrogens [8, 49, 50]. Ochoa et al. observed also the rapid and transient increase of VEGF in rat anterior pituitary following E2 administration [49]. Estrogen treatment augmented also the VEGF receptor 2 (flk-1/KDR) expression and induced neovascularisation as well as the growth and enlargement of blood vessels in the rat pituitary [9, 51].

Similarl to the former data, we observed a significant elevation of the VEGF-immunopositive anterior pituitary cells number and blood vessels density after 8-day exposure of rats to DES. In the pituitaries of estrogen-treated animals, the VEGF-positive cells were distributed in patches surrounding the blood vessels and scattered through all the glandular pituitary (Figure 5(b)). In contrast, only weak staining signals with antibodies to VEGF were detected in a separate, diffusely distributed pituitary cells of untreated control animals (Figure 5(a)). Similar results were obtained by Banerjee et al., who revealed a strong increase in both the intracellular VEGF protein expression and in the VEGF-positive blood vessel density in rat pituitary after 7 days of E2 exposure [9]. At the same time, we noticed a significant fall in the number of VEGF-immunostained cells as well as blood vessels in the pituitaries of rats receiving the specific AT1 receptor antagonist losartan. Treatment with ACE inhibitor captopril or AT2 receptor antagonist PD123319 led to the decline in VEGF-positive blood vessel density, whereas it did not affect the VEGF immunostaining in anterior pituitary cells. These results are compatible with our earlier data, showing that losartan is more effective in inhibition of the estrogen-induced pituitary vascular changes than AT2 blocker PD 123319 [52].

The inhibitory effect of losartan suggests that AT1 receptors are involved in the control of VEGF expression, in both pituitary cells and vessels. In contrast, the effects of PD123319, a selective AT2 receptor blocker, and of captopril, an ACE inhibitor, on VEGF expression seem to be confined exclusively to the blood vessels. Recently, it was shown that in human pituitary adenomas AT2 receptor immunopositivity is absent in adenoma cells but it is very strong in blood vessel walls [53]. If the same vascular localization of AT2 receptors concerns also the rat anterior pituitary, this could explain the selective effects of PD123319 on vascular VEGF. The selective effect of captopril on VEGF immunopositivity in blood vessels is more difficult to explain, since this agent inhibits ang II production, and the latter is an agonist of both AT1 and AT2 receptors. However, it should be pointed that inhibition of ang II synthesis leads to the enhanced synthesis of smaller angiotensin peptides via alternative enzymatic pathways [54]. Since the products of ang II degradation have been shown to induce VEGF secretion by pituitary cells, their overproduction possibly offsets the decline of VEGF production resulting from inhibited ang II synthesis. Moreover, concomitant influences of ACE inhibitor on the activity of various enzymes, different from ACE, should be taken into consideration. ACE inhibitors are established to induce kininase II, increasing thereby the tissue bradykinin concentration and affecting angiogenesis via endothelial NO release. Thanks to the presence the thiol radical, captopril is also able to exert the direct influence on vascular growth [55, 56].

Regardless the direct proangiogenic activities of angiotensin, the indirect effects of RAS and its inhibitors on the anterior pituitary angiogenesis should not be excluded. The full-length PRL molecule has been shown to possess angiogenic effect in an *in vivo* assays using the chick chorioallantoic membrane (CAM) [57]. On the other hand, estrogens belong to the potent stimuli of PRL secretion, whereas both ACE inhibitors and angiotensin receptor antagonists have been proved to suppress the DES-induced release of PRL [28]. In this context we can not exclude that inhibition of PRL release following the administration of captopril, losartan, or PD123319 may response, at least in part, for the decrease in number of VEGF-positive vessels in rat anterior pituitary. The identification of mechanisms engaged in the RAS-dependent regulation of angiogenesis in the pituitary gland needs further studies.

The involvement of RAS in regulation of the VEGF-dependent angiogenesis has already been reported with respect to various tissues [58–62]. This is also worth mentioning that both the AT1 and AT2 receptor has been found to participate in proangiogenic effects of angiotensins. The AT1 receptor subtype was implicated in the regulation of angiogenesis during skeletal muscle regeneration, the increase of vessel density in cremaster muscle of rats, the induction of VEGF receptor expression in bovine retinal microcapillary endothelial cells, and the VEGF-dependent angiogenesis in the Matrigel model in mice [63–66]. At the other hand, a higher VEGF expression in the retina of experimental diabetic rats and ang-II-treated rats was partially determined by AT2 receptor activation [67]. The AT2 receptor blockade resulted in an important decrease of the VEGF and its receptor KDR expression in kidney of ang-II-infused rats [68]. Furthermore, the AT2 receptor blockade using PD123319 in animal *in vivo *models of fibrosarcoma and LL/2 cells carcinoma delayed tumorigenesis by inhibiting malignant cell proliferation and angiogenesis as well [69]. Similar effects were obtained with respect to the anterior pituitary cells. In earlier *in vitro* study, losartan and PD123319 abrogated the stimulatory influence of ang II on VEGF secretion in GH3 cell culture [31]. Furthermore, the antagonists of both the AT1 and AT2 receptors were also demonstrated to prevent the DES-induced enlargement of blood vessels in anterior pituitary in rodents [52]. Taken together, the present and the previous results imply, that RAS mediates estrogen-induced vascular growth in anterior pituitary, and this process involves the induction and secretion of VEGF via AT1 and AT2 receptors.

 Summing up, our results suggest the involvement of RAS in DES-induced cellular proliferation and VEGF expression in anterior pituitary of rat. Both AT1 and AT2 receptors appear to mediate the observed mitogenic and proangiogenic effects of estrogens. Since cellular proliferation and blood vessel formation are indispensable for the tumor development, our study may suggest the participation of RAS in pathogenesis of estrogen-induced PRL-secreting adenoma. Further studies are necessary to extend the reported above properties of RAS antagonists of the to human lactotropic cells.
